# Supplementary material for: Rejuvenation as the origin of planar defects in the CrCoNi medium entropy alloy
Source: Nat Commun. 2024 Feb 16;15:1402. doi: 10.1038/s41467-024-45696-z (PMC10873362; doi:10.1038/s41467-024-45696-z)
Supplement: Supplementary file 1 — Supplementary Information [file 41467_2024_45696_MOESM1_ESM.pdf]

Supplementary materials

Rejuvenation as the origin of planar defects in the CrCoNi medium entropy alloy

Yang Yang[1,2,*], Sheng Yin[3], Qin Yu[3], Yingxin Zhu[2], Jun Ding[4], Ruopeng Zhang[5], Colin Ophus[1], Mark Asta[3,5] , Robert O. Ritchie[3,5] , Andrew M. Minor[1,5,*]

[1] National Center for Electron Microscopy, Molecular Foundry, Lawrence Berkeley National Laboratory, Berkeley, CA, USA.

[2] Department of Engineering Science and Mechanics and Materials Research Institute, The Pennsylvania State University, University Park, PA, USA

[3] Materials Sciences Division, Lawrence Berkeley National Laboratory, Berkeley, CA, USA

[4] Center for Alloy Innovation and Design (CAID), State Key Laboratory for Mechanical Behavior of Materials, Xi'an Jiaotong University, Xi'an, China

[5] Department of Materials Science and Engineering, University of California, Berkeley, CA, USA.

Table of Contents

Supplementary Figures. **2**

Supplementary Note 1. .... **10**

Supplementary References. **12**

Supplementary Figures.

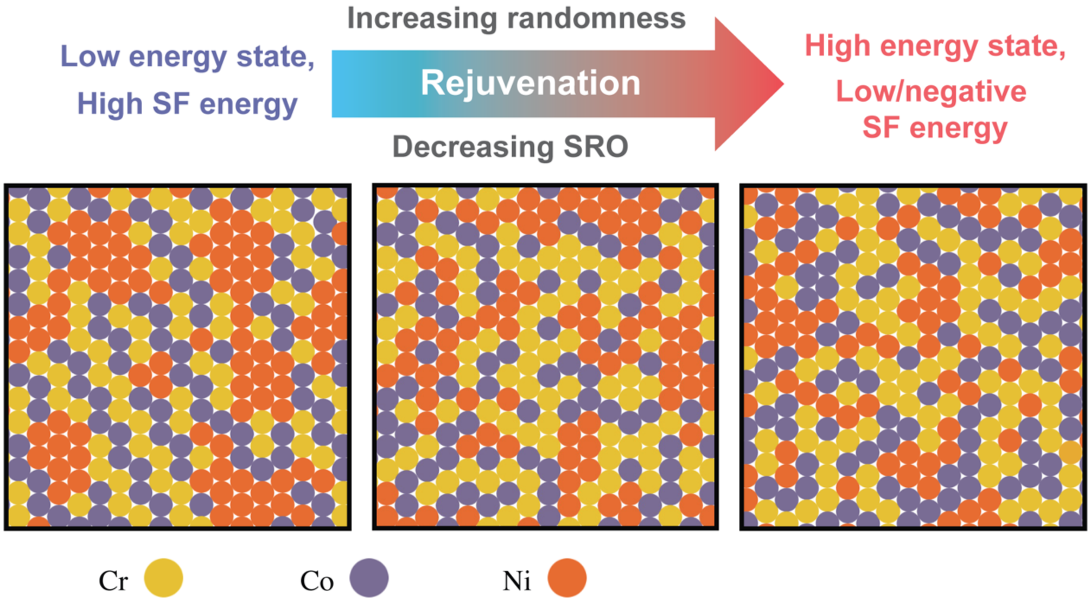

Supplementary Figure 1. Schematic drawing showing the implication of rejuvenation in high or medium- entropy alloys (HEA/MEAs). The reduction of short-range order (SRO) leads to rejuvenation. As a result, the system has a higher energy, while the stacking fault (SF) energy is lower (positive) or becomes more negative. The atomic arrangement is sketched using the results from a molecular dynamics modeling[11] as a reference, which is an open-access paper under the Creative Commons licence (http://creativecommons.org/licenses/by/4.0/).

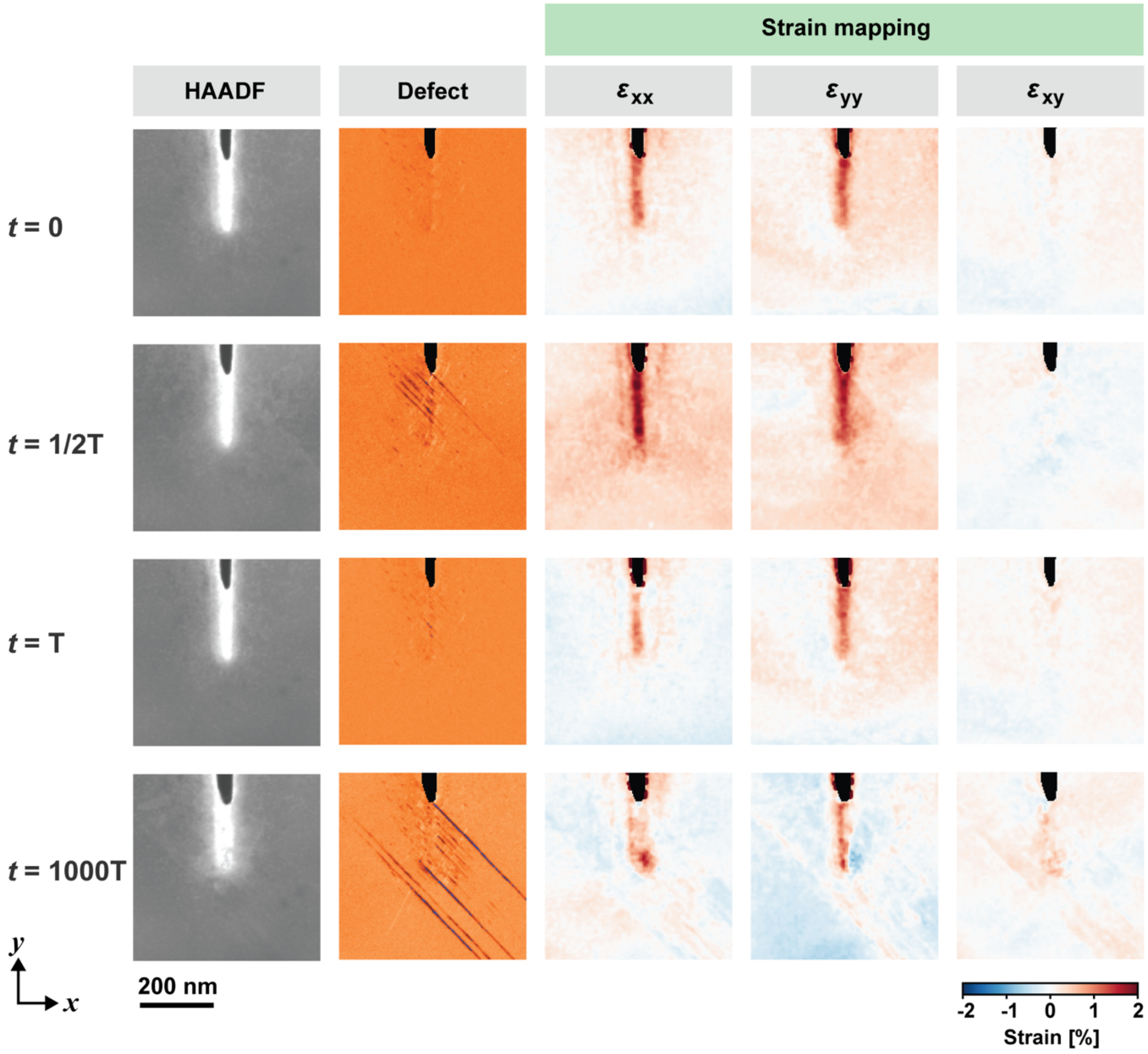

Supplementary Figure 2. High-angle annular dark-field (HAADF) scanning transmission electron microscopy (STEM) and four-dimensional STEM (4D-STEM) characterization of the CrCoNi MEA sample at different stages of deformation. *t* is time. T is the length of time per cycle. ε_{xx}, ε_{yy}, and ε_{xy} are the normal strain along *x* direction, normal strain along *y* direction, and shear strain, respectively.

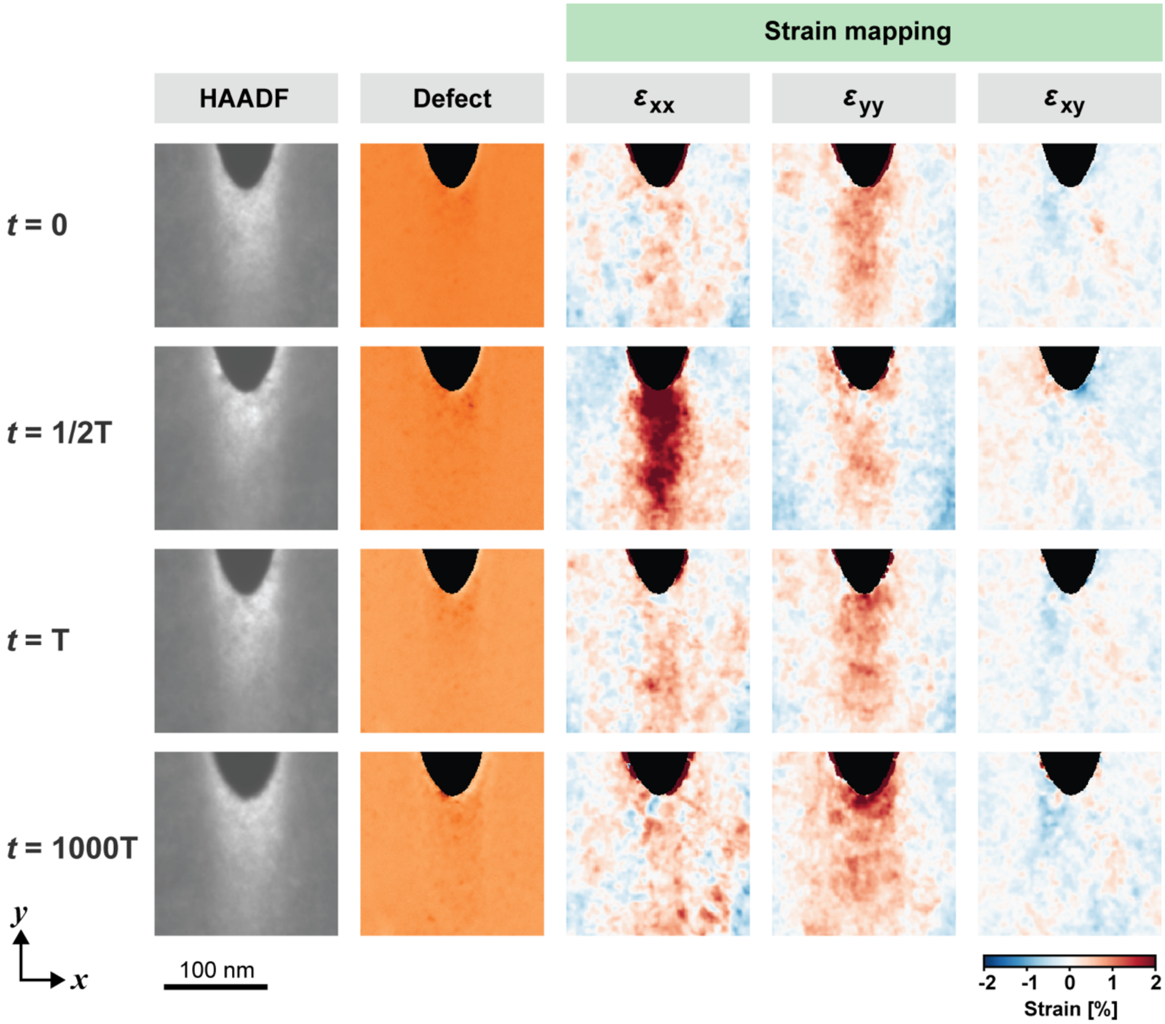

Supplementary Figure 3. STEM-HAADF and 4D-STEM characterization of the pure Ni sample at different stages of deformation. t is time. T is the length of time per cycle. ε_{xx}, ε_{yy}, and ε_{xy} are the normal strain along x direction, normal strain along y direction, and shear strain, respectively.

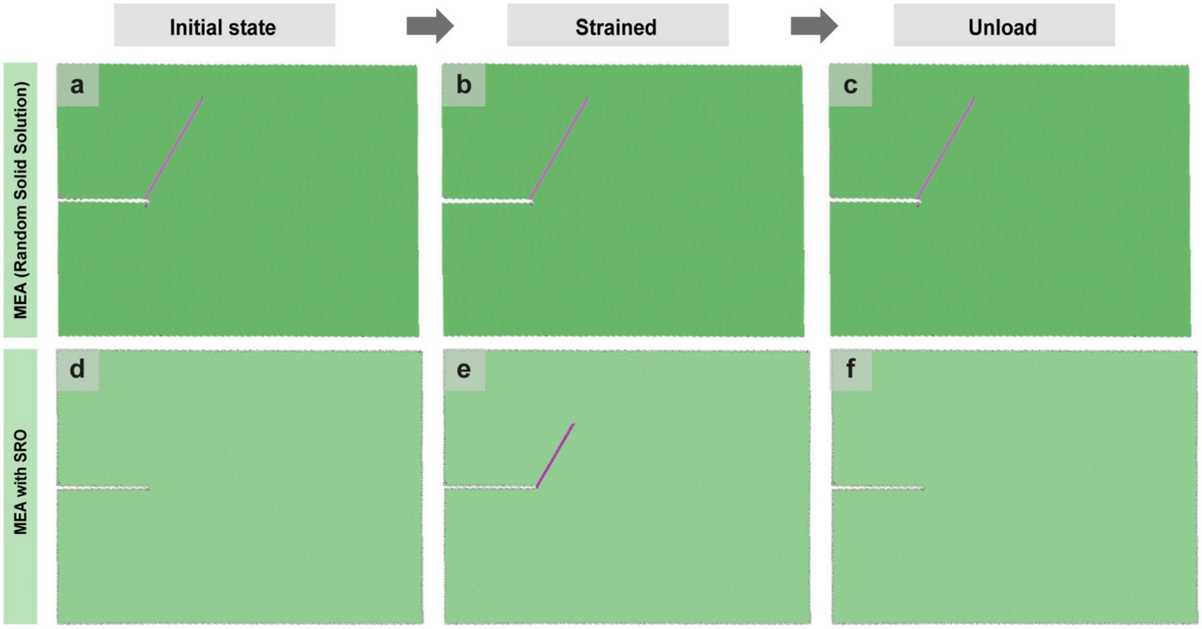

Supplementary Figure 4. MD simulation to reveal the reversibility of SF in MEA during a single loading cycle. The FCC and HCP phases are colored green and red, respectively. | **a-c.** Snapshots showing SF is irreversible if the MEA is random solid solution (RSS). **d-f.** Snapshots showing SF is reversible if the MEA contains SRO. The FCC and HCP phases are colored green and magenta, respectively.

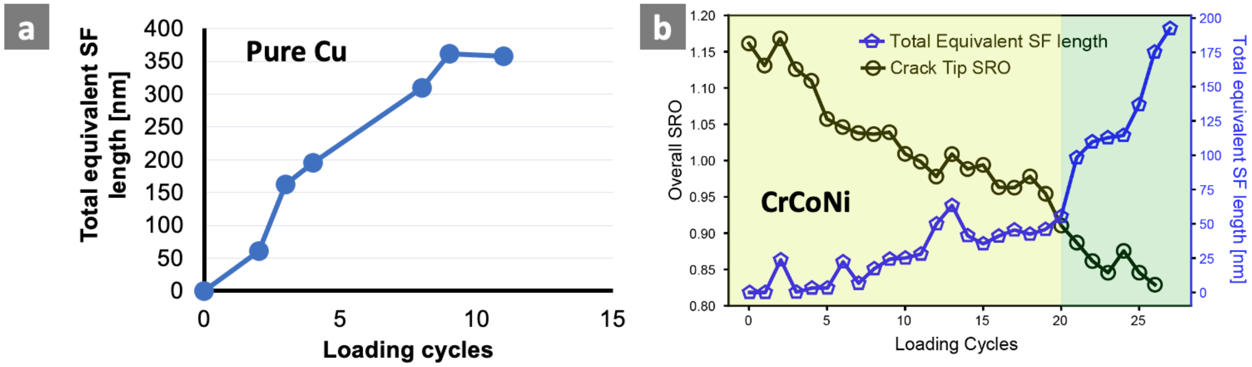

Supplementary Figure 5. Comparative analysis of stacking fault evolution under cyclic loading in various metals. | **a.** Pure Cu. The depicted curve is derived from our analysis of the molecular dynamics simulation snapshots provided in Reference[18]. **b.** The equiatomic CrCoNi MEA investigated in the current study, as originally depicted in Fig. 4d of this manuscript. **Note:** The comparison between parts **a** and **b** is inherently qualitative. The analysis for part **a** is based on interpreted images from Reference[18], rather than direct raw data, and the simulations in **a** and **b** were performed on samples with different crack shapes, which may introduce certain inaccuracies.

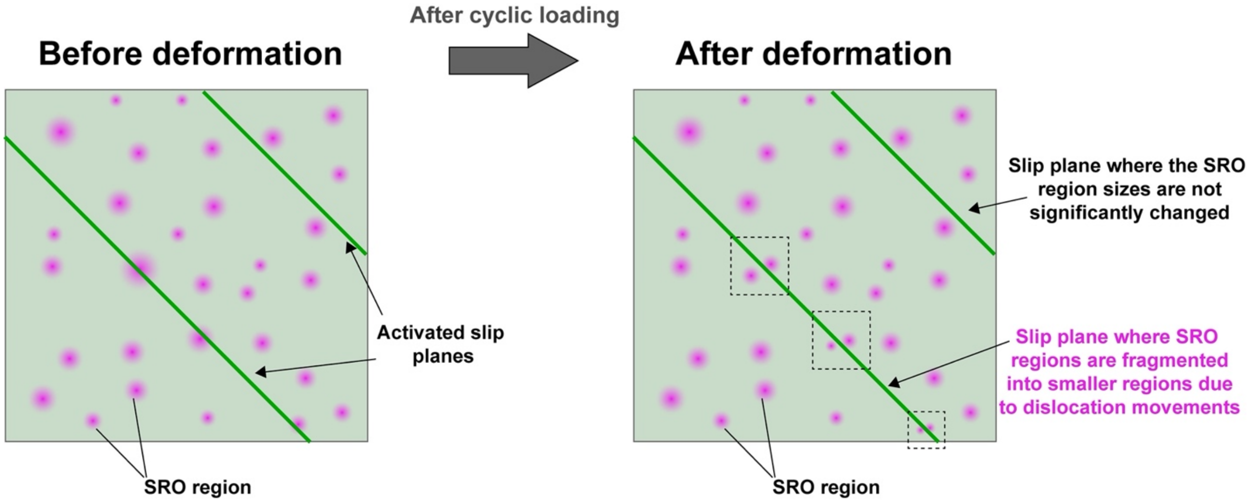

Supplementary Figure 6. Schematic drawing showing the influence of dislocation glide on SRO region density and size.

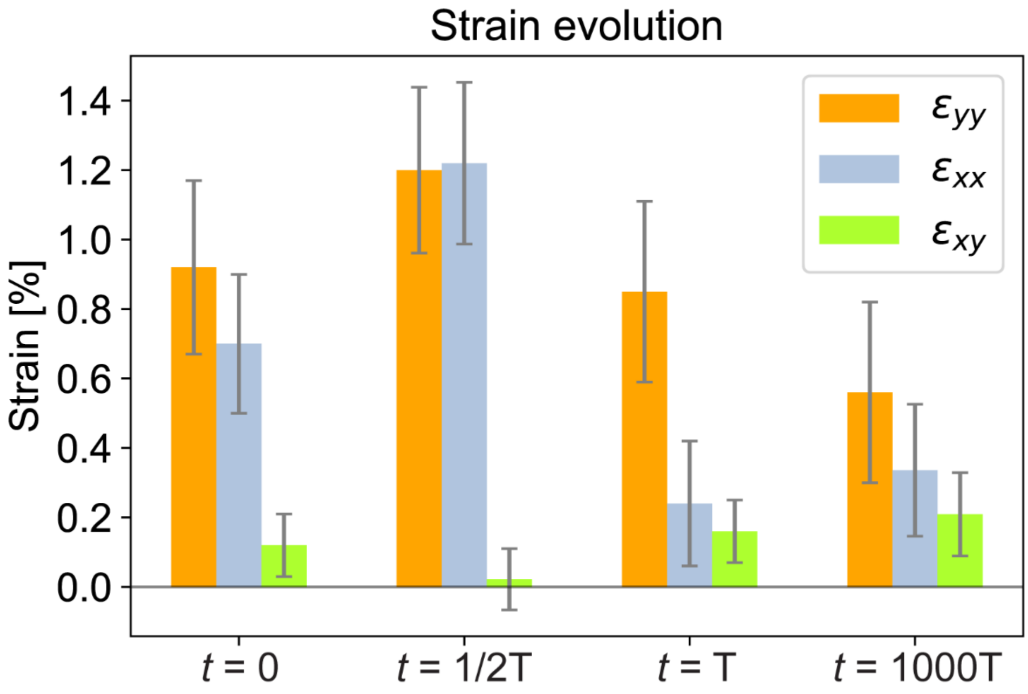

Supplementary Figure 7. 4D-STEM characterization of the average strain in a squared region ahead of the crack tip in the CrCoNi MEA sample after 1000 cycles of deformation. The error bars show the standard deviation. *t* is time. T is the length of time per cycle. ε_{xx}, ε_{yy}, and ε_{xy} are the normal strain along *x* direction, normal strain along *y* direction, and shear strain, respectively.

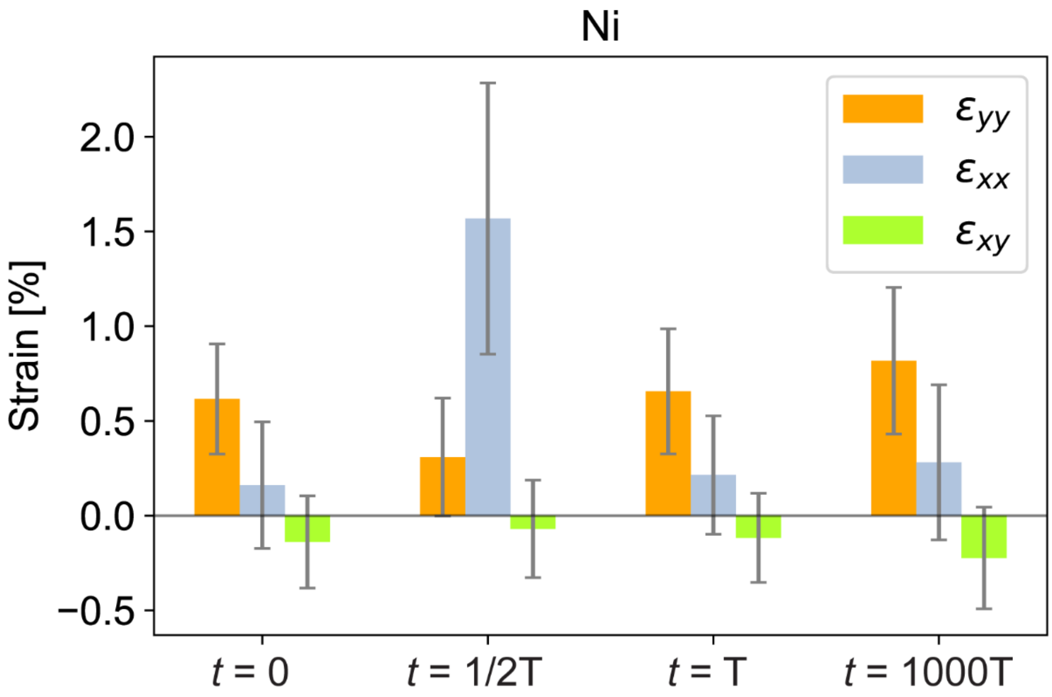

Supplementary Figure 8. 4D-STEM characterization of the average strain in a squared region ahead of the crack tip in the Ni sample after 1000 cycles of deformation. The error bars show the standard deviation. t is time. T is the length of time per cycle. ε_{xx}, ε_{yy}, and ε_{xy} are the normal strain along x direction, normal strain along y direction, and shear strain, respectively.

Supplementary Note 1.

While a comprehensive study of how vacancies interact with defects during cyclic loading is beyond the scope of this work, we hope to briefly touch upon the mechanisms at play that are relevant to this study, *i.e.*, how vacancies generated during cyclic loading can influence short-range order (SRO) evolution even at room temperature.

1) Vacancy facilitates the formation of SRO.
   During cyclic loading, vacancies will be emitted from the crack tip[1] directly, or generated by the back-and-forth dislocation movement even at room temperature[2]. These excess vacancies can facilitate diffusion. Based on the modeling[3] by Li et al, the degree of SRO is higher when the annealing temperature is lower. Thus, at room temperature, the thermal dynamic driving force for SRO formation is very high. However, SRO cannot reach this high SRO value predicted at its equilibrium at room temperature because the kinetics is typically too slow. The excess vacancy led by cyclic loading may facilitate SRO formation due to more rapid kinetics.

   Previously, people have studied how precipitates or nanoclusters interact with vacancies and dislocations during cyclic loading, such as in aluminum alloys[2]. For example, based on a classical (Zener) model for diffusion-controlled growth of spherical particles, the growth rate of particles has an inverse relationship with the strain rate. As shown in previous studies[2], the growth rate of local clusters during cyclic deformation comprises a destructive term tied to dislocations and a constructive term associated with vacancy-mediated mechanisms, the latter being highly sensitive to the strain rate, as shown by the equation below[2]:

$$\frac{\mathrm{dR}}{\mathrm{d}\epsilon} = \frac{\mathrm{dR}^{+}}{\mathrm{d}\epsilon} + \frac{\mathrm{dR}^{-}}{\mathrm{d}\epsilon} \tag{1}$$

   where R and ϵ are the size of the spherical particle and the strain, respectively. For the Zener model, the gain term can be:

$$\frac{\mathrm{dR}^{+}}{\mathrm{d}\epsilon} = \frac{1}{\dot{\epsilon}}\frac{\mathrm{dR}}{\mathrm{dt}} = \frac{1}{\dot{\epsilon}}\left[\frac{\mathrm{D}\cdot(\mathrm{C_b}-\mathrm{C_{eq}})}{\mathrm{R}\cdot(\mathrm{C_p}-\mathrm{C_{eq}})}\right]\frac{C_{ex_v}}{C_{eq_v}} \tag{2}$$

   where C_b, C_p, C_{eq}, C_{eq_v}, C_{ex_v}, t, $\dot{\epsilon}$, and D are bulk solute content, solution concentration in the precipitate, equilibrium solute concentration, equilibrium vacancy concentration, excess concentration of vacancy, time, strain rate and diffusivity of the solute in the presence of an equilibrium concentration of vacancies, respectively.

   While the model for spherical precipitate or local cluster may not be directly applied for the SRO, it was hypothesized that the mechanisms might be similar. The vacancy-mediated SRO

formation, though crucial in systems at lower strain rates, gets significantly diminished in high-strain-rate scenarios.

At lower strain rates and over extended cycling periods, the influence of vacancies becomes more pronounced. These excess vacancies may decelerate the dislocation-mediated destruction of SRO or potentially enhance the degree of SRO. Consequently, dislocation motion is expected to become more sluggish due to more SROs acting as an impediment. Furthermore, with a higher degree of SRO, the SF energy increases, resulting in fewer long and irreversible SFs. This leads to a tendency for SFs to be more transient and reversible in nature.

For our system, given the high melting temperature (1690 K) of CrCoNi MEA[4], the homologous temperature is low (T/T_m = 0.178), and the diffusion processes are inherently sluggish. Thus, the vacancy-mediated SRO formation is considered minimal and possibly negligible. Indeed, our observations indicate a continuous reduction in SF energy throughout our experiments, which suggests a diminishing degree of SRO. This trend points to the predominance of dislocation-induced SRO destruction. Additional evidence is the electric resistivity[5] studies on CrCoNi MEA under single-cycle tensile loading at a similar strain rate, which also shows the dominance of SRO reduction rather than SRO formation.

2) Other effects of vacancies and vacancy clusters on dislocation include altering dislocation mobility, facilitating dislocation climb, and lock or pinning of dislocation, etc. At room temperature, these interactions are generally less dynamic than at higher temperatures, owing to reduced atomic mobility.

Supplementary References.

1. Nishimura, K. & Miyazaki, N. Molecular dynamics simulation of crack growth under cyclic loading. *Comput. Mater. Sci.* **31**, 269–278 (2004).
2. Sun, W. *et al.* Precipitation strengthening of aluminum alloys by room-temperature cyclic plasticity. *Science* **363**, 972–975 (2019).
3. Li, Q. J., Sheng, H. & Ma, E. Strengthening in multi-principal element alloys with local-chemical-order roughened dislocation pathways. *Nat. Commun.* **10**, 1–11 (2019).
4. Wu, Z., Bei, H., Pharr, G. M. & George, E. P. Temperature dependence of the mechanical properties of equiatomic solid solution alloys with face-centered cubic crystal structures. *Acta Mater.* **81**, 428–441 (2014).
5. Li, L. *et al.* Evolution of short-range order and its effects on the plastic deformation behavior of single crystals of the equiatomic Cr-Co-Ni medium-entropy alloy. *Acta Mater.* **243**, (2023).
