# Supplementary material for: Rejuvenation as the origin of planar defects in the CrCoNi medium entropy alloy
Source: Nat Commun. 2024 Feb 16;15:1402. doi: 10.1038/s41467-024-45696-z (PMC10873362; doi:10.1038/s41467-024-45696-z)
Supplement: Supplementary file 3 — Description of Additional Supplementary Information [file 41467_2024_45696_MOESM3_ESM.pdf]

Description of Additional Supplementary Files

File Name: Supplementary Movie 1
Description: Molecular dynamics simulation showing that the stacking faults are irreversible during a single cycle loading in a CrCoNi medium entropy alloy containing no short-range order before loading. The FCC and HCP phases are colored green and magenta, respectively.

File Name: Supplementary Movie 2
Description: Molecular dynamics simulation showing that the stacking faults are reversible during a single cycle loading in a CrCoNi medium entropy alloy containing short-range order before loading. The FCC and HCP phases are colored green and magenta, respectively.

File Name: Supplementary Movie 3
Description: Molecular dynamics simulation showing that the evolution of stacking faults during cyclic loadings in a CrCoNi medium entropy alloy containing short-range order before loading. The FCC and HCP phases are colored green and magenta, respectively.
